# Efficient Parvovirus Replication Requires CRL4^Cdt2^-Targeted Depletion of p21 to Prevent Its Inhibitory Interaction with PCNA

**DOI:** 10.1371/journal.ppat.1004055

**Published:** 2014-04-03

**Authors:** Richard O. Adeyemi, Matthew S. Fuller, David J. Pintel

**Affiliations:** Department of Molecular Microbiology and Immunology, C.S. Bond Life Sciences Center, University of Missouri-Columbia, School of Medicine, Columbia, Missouri, United States of America; Wake Forest University, United States of America

## Abstract

Infection by the autonomous parvovirus minute virus of mice (MVM) induces a vigorous DNA damage response in host cells which it utilizes for its efficient replication. Although p53 remains activated, p21 protein levels remain low throughout the course of infection. We show here that efficient MVM replication required the targeting for degradation of p21 during this time by the CRL4^Cdt2^ E3-ubiquitin ligase which became re-localized to MVM replication centers. PCNA provides a molecular platform for substrate recognition by the CRL4^Cdt2^ E3-ubiquitin ligase and p21 targeting during MVM infection required its interaction both with Cdt2 and PCNA. PCNA is also an important co-factor for MVM replication which can be antagonized by p21 *in vitro*. Expression of a stable p21 mutant that retained interaction with PCNA inhibited MVM replication, while a stable p21 mutant which lacked this interaction did not. Thus, while interaction with PCNA was important for targeting p21 to the CRL4^Cdt2^ ligase re-localized to MVM replication centers, efficient viral replication required subsequent depletion of p21 to abrogate its inhibition of PCNA.

## Author Summary

Many DNA viruses induce and then exploit host cellular DNA damage responses to generate a suitable environment for their continued replication. Parvoviruses, important disease agents in both humans and animals, rely on host DNA polymerases to replicate their genomes in cell-cycle arrested cells. We show that efficient parvovirus replication requires the recruitment to viral replication compartments of a host cellular E3-ubiquitin ligase, CRL4^Cdt2^, to target the potent cell cycle regulator p21 for subsequent degradation. The DNA polymerase-δ cofactor PCNA provides a molecular platform for initial substrate recognition by this ligase, and subsequent p21 depletion prevents its continued interaction with PCNA which otherwise inhibits efficient viral replication. Virally-induced p21 degradation represents another way of promoting efficient replication of DNA polymerase-δ-dependent viruses.

## Introduction

Minute Virus of Mice (MVM) is an autonomously-replicating parvovirus which induces a DNA damage response resulting in substantial p53 activation which persists throughout the course of viral replication [1]. p53 is a well-established activator of p21^WAF1/Cip1^ (hereafter referred to as p21) expression. Transient expression of the MVM NS1 protein alone also leads to an increase in p21 levels [2], [3]. However, surprisingly, while these signals lead to an increase in p21 RNA accumulation, p21 protein levels remain low throughout the course of infection, including during the prolonged G2 phase in which the viral genome is replicated [2]. p21 can be a potent antiviral factor and possesses several potentially inhibitory activities including cyclin-dependent kinase (CDK) inhibition and repression of E2F1-mediated expression [4]. In addition, p21 has been shown to be an effective inhibitor of the DNA polymerase δ cofactor PCNA [5]–[7], and it has been shown to inhibit MVM replication *in vitro* [8]. p21 depletion during MVM infection was shown to be proteasomally mediated, suggesting that an E3 ubiquitin ligase was involved in targeting p21 for degradation [2].

Viruses often make use of the ubiquitin conjugation machinery to target for degradation cellular proteins that might otherwise negatively affect viral replication [9]. The Cullin-RING Ligase (CRL) CRL4^Cdt2^ consists of the scaffold protein Cullin 4 and the homo-trimeric protein DDB1 which serves as an adaptor for the putative substrate recognition protein Cdt2. This ligase has been shown to program the ubiquitination and subsequent degradation of p21 in response to DNA damaging agents such as UV treatment in order to ensure low p21 levels during S-phase [10]–[12]. Upon DNA damage or S-phase entry CRL4^Cdt2^ is recruited to chromatin *via* PCNA interaction where it targets substrate proteins for degradation [13].

We show here that efficient MVM replication in S/G2 arrested cells required the targeting for proteasomal degradation of p21 by the CRL4^Cdt2^ E3-ubiquitin ligase which was re-localized to viral chromatin within active MVM replication centers. PCNA provides a molecular platform that aids substrate recognition by the CRL4^Cdt2^ E3-ubiquitin ligase, and p21 targeting to this ligase during MVM infection required its interaction with PCNA. PCNA is also an important co-factor for DNA polymerase δ-dependent MVM replication which can be antagonized by p21 *in vitro*. Expression of a stable p21 mutant that retained interaction with PCNA inhibited MVM replication, while a stable p21 mutant which could no longer interact with PCNA did not. Introduction of a p21-derived peptide that bound to PCNA also substantially decreased viral replication. Our results suggest that interaction with PCNA was important for targeting p21 to the re-localized CRL4^Cdt2^ ubiquitin ligase, yet subsequent depletion of p21 was required to prevent its sustained interaction with PCNA which otherwise inhibited efficient viral replication.

## Results

### The CRL4^Cdt2^ ligase mediates p21 degradation during MVM infection

**Figure 1:**
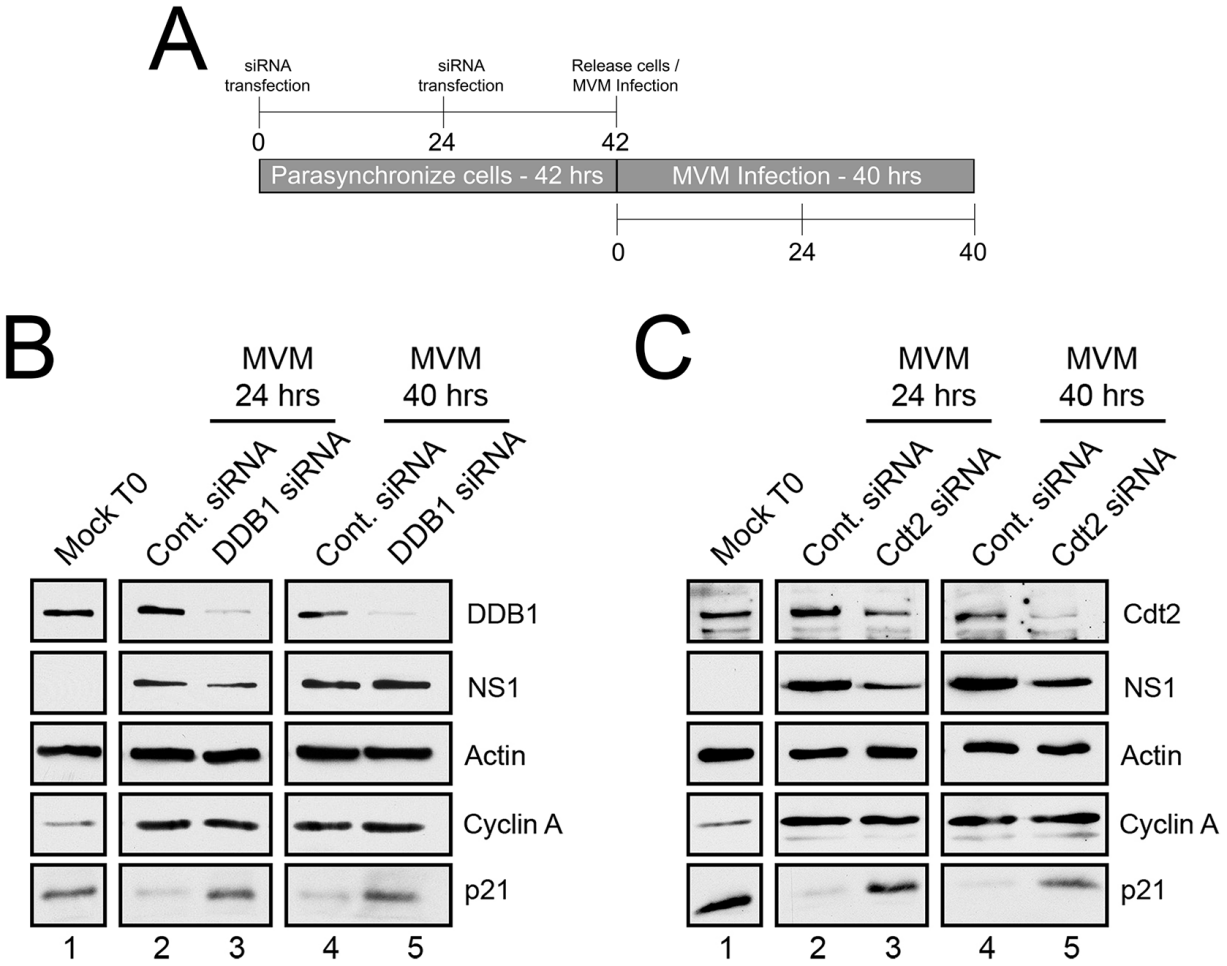
p21 degradation is mediated by the CRL4^Cdt2^ ligase complex. **A**) Schematic illustrating the experimental protocol for siRNA knockdown of ligase components in Figures 1B and 1C. **B and C**) *p21 degradation requires DDB1 (B) and Cdt2 (C)*. Murine A9 cells were targeted with control siRNA or siRNA to DDB1 (B) or Cdt2 (C) as depicted in Figure 1A. Uninfected control cells were harvested at the time of release (Mock T0). Infections were done at the time of release at an MOI of 10 before harvest at 24 and 40 hr pi. Western blots were performed using antibodies against the indicated proteins.

The CRL4^Cdt2^ E3 ubiquitin ligase has been implicated in targeting p21 for proteasomal degradation upon S-phase entry and after cellular DNA damage [10]–[12]. Was this ubiquitin ligase also enlisted to target p21 at late times during MVM infection when cells were blocked at the G2/M border? To test this possibility, DDB1 and Cdt2, components of this ligase which are not present in other E3 ubiquitin ligases known to modify p21 [13], [14], were targeted *via* RNAi in the protocol illustrated in Figure 1A.

For these experiments cells were parasynchronized prior to infection to maximize the number of cells progressing uniformly through S-phase. This both synchronized infection and the characterization of p21 depletion. At the time of release from the synchronization procedure (as the cells progressed from G0 to G1) there were high levels of p21 expression (Mock T0, Figures 1B and 1C, lanes 1), which were reduced 24 hr post MVM infection (Figures 1B and 1C, lanes 2), and remained low up to 40 hr pi (Figures 1B and 1C, lanes 4). Targeting of endogenous DDB1 (panel 1B) or Cdt2 (panel 1C) by RNAi, which led to significant depletion of these proteins (Figure 1B and 1C, lanes 3 and 5), substantially prevented the loss of p21 both at 24 hr pi (Figures 1B and 1C, lanes 3), and also 40 hr pi (Figure 1B and 1C, lanes 5) – the latter being a point well past S-phase when infected cells are known to be arrested in G2 [1]. Expression of cyclin A indicated unperturbed entry into S-phase (Figures 1B and C, lanes 2–5), and expression of the viral NS1 protein indicated that the initiation of viral infection was unaffected by the RNAi protocol (Figures 1B and C, lanes 2–5). Similar results were also obtained following knockdown of Cullin 4A, a component of the CRL4^Cdt2^ ligase also present in several other ubiquitin ligases (data not shown). Taken together, these results indicated that the CRL4^Cdt2^ ligase was responsible for p21 degradation throughout virus infection.

CRL4^Cdt2^ also targets Set8 and Cdt1 for degradation during S-phase and in response to DNA damaging agents [15]–[17]. We also observed loss of Set8 in response to MVM infection (Figure S1). Cdt1 levels were not reduced for reasons not yet clear.

### CRL4^Cdt2^ ligase function is required for efficient MVM replication

**Figure 2:**
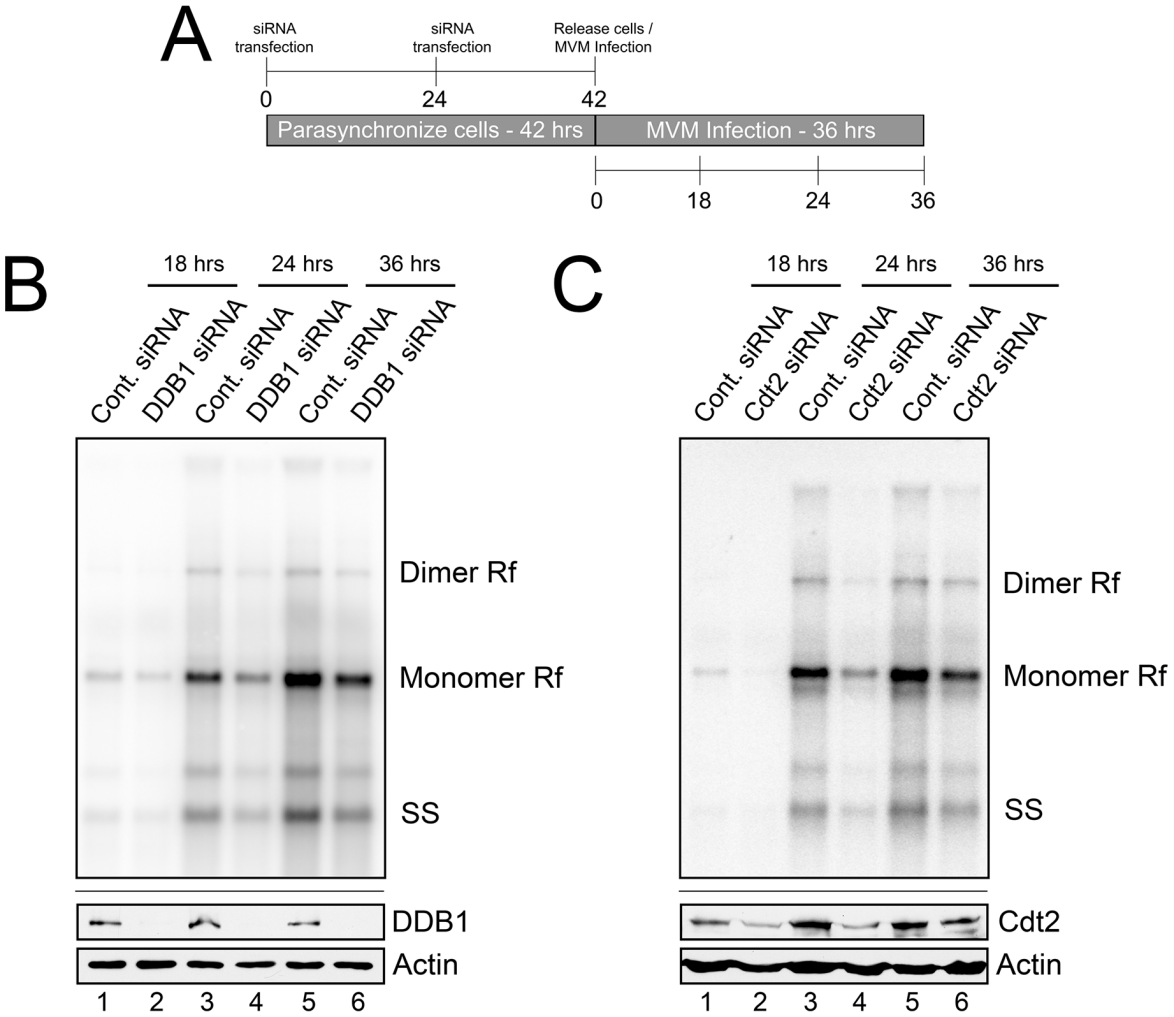
The CRL4^Cdt2^ E3 ligase complex is important for MVM replication. **A**) Schematic showing the experimental protocol for figures 2B and 2C. **B and C**) *Upper panels:* murine A9 cells treated with siRNA as shown in 2A were infected at an MOI of 0.5, harvested at the indicated time points and processed for Southern blotting using an MVM genomic probe. Rf - replicative forms. SS - single stranded genomic DNA. Representative Southern Blots are shown; quantifications in the text reflect two DDB1 and three Cdt2 separate knockdown experiments. *Lower panels:* western blots show knockdown of DDB1 and Cdt2 done in parallel experiments under identical conditions to replication assays.

We next examined the functional consequence of CRL4^Cdt2^ E3 ligase depletion for viral replication using the protocol illustrated in Figure 2A. DDB1 knockdown resulted in an approximate 2-fold decrease in accumulated viral replicative forms at each time point compared to application of negative control siRNA (Figure 2B, compare lanes 1 to 2, 3 to 4, 5 to 6). Cdt2 knockdown during infection resulted in an approximate 2.5-fold decrease in accumulated viral replicative forms at each time point when compared to negative control siRNA (Figure 2C, compare lanes 1 to 2, 3 to 4, 5 to 6). Importantly, as mentioned above, expression of NS1 (Figure 1), and flow cytometric analysis (Figure S2), confirmed normal entry into S-phase following this synchronization and RNAi protocol which thus did not affect the S-phase dependent initiation of infection. These results demonstrated that the activity of the CRL4^Cdt2^ E3 ubiquitin ligase was necessary for efficient viral replication.

### The CRL4^Cdt2^ ligase is recruited to viral replication compartments

**Figure 3:**
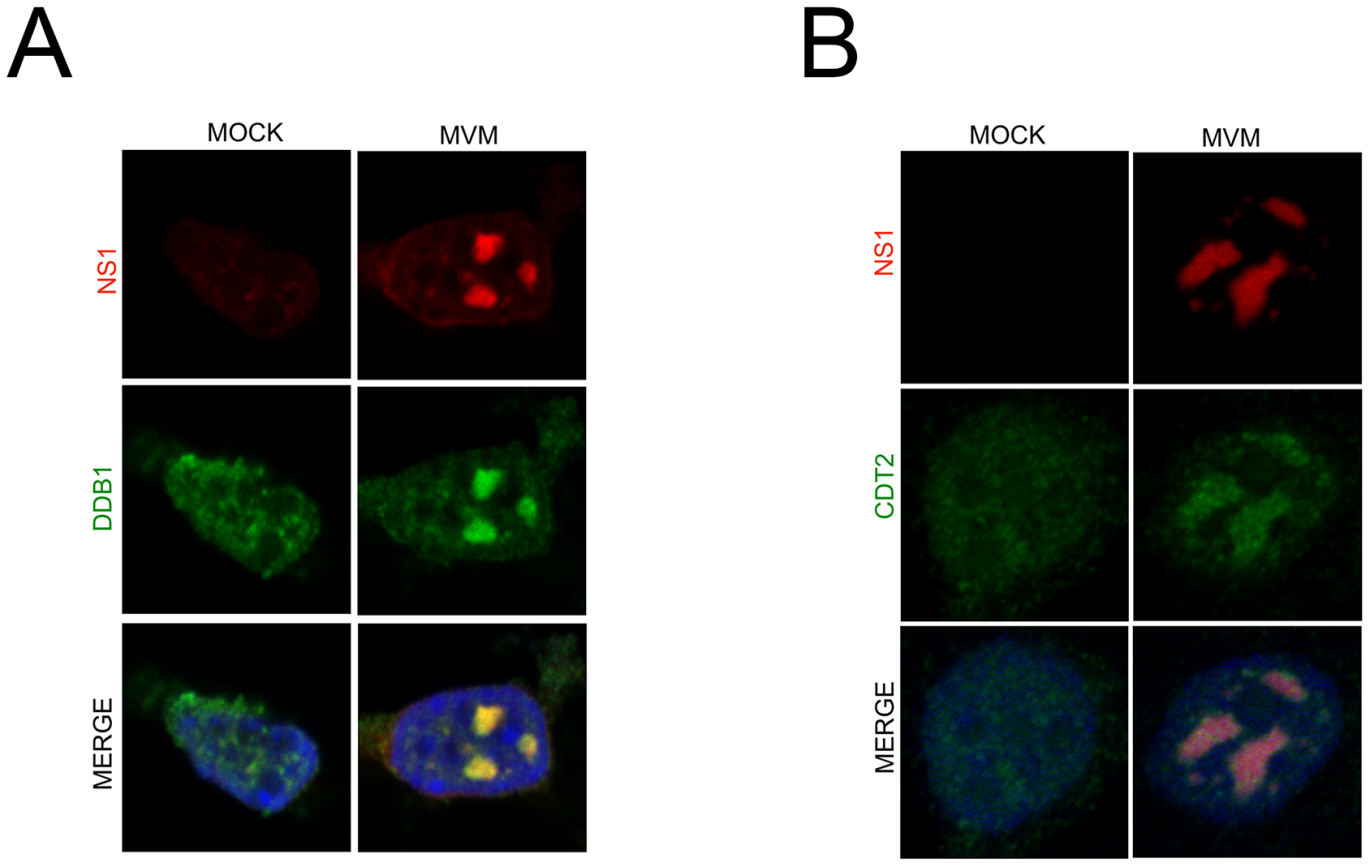
The CRL4^Cdt2^ ligase is recruited to viral replication compartments. **A and B**) Murine A9 cells were mock infected or infected with MVM at an MOI of 10. At 24 hr pi cells were pre-extracted using cytoskeletal buffer and processed for IF using antibodies against NS1 and DDB1 (A) or Cdt2 (B).

MVM replicates in nuclear compartments termed autonomous parvovirus associated replication (APAR) bodies which have been shown to be enriched for cellular proteins such as DNA polymerase δ, RPA, cyclin A, and PCNA, which are also essential for parvovirus replication [18]. These nuclear bodies have been shown, *via* BrdU staining, to serve as sites of ongoing viral replication in infected cells and can be visualized by staining for the viral replicator protein NS1 [19]. Importantly, whereas punctate staining distributed throughout the nucleus was observed in mock treated cells, we detected recruitment of both DDB1and Cdt2 to NS1-containing viral replication compartments. Resistance to detergent pre-extraction prior to immunofluorescence (Figure 3A and 3B, note the merged images for each) also suggested that it may be bound to viral chromatin. These results indicated that MVM infection redirected the viral replication-enhancing CRL4^Cdt2^ ligase complex to APAR bodies. The recruitment was specific for the CRL4^Cdt2^ ligase because the APC/C^Cdc20^ E3 ligase, which targets p21 for degradation after mitotic entry [20], was not similarly recruited (Figure S3). This is the first demonstration of the specific recruitment of a cellular E3 ubiquitin ligase to parvoviral replication compartments.

### Depletion of p21 during MVM infection requires its interaction with the CRL4^Cdt2^ ligase and PCNA

The DNA polymerase δ cofactor PCNA is known to target the CRL4^Cdt2^ E3 ubiquitin ligase to cellular chromatin *via* its interaction with Cdt2 during normal cell division [13], [21]. Ubiquitin modification of p21 by the CRL4^Cdt2^ ligase requires Cdt2 interaction with the p21 degron motif, as well as interaction between p21 and PCNA *via* its PCNA-interaction (PIP) box [22]. p21 is recruited to UV-induced DNA lesions *via* interaction with PCNA, and a p21 mutant defective in binding to PCNA was resistant to degradation following UV treatment [10], [12].

**Figure 4:**
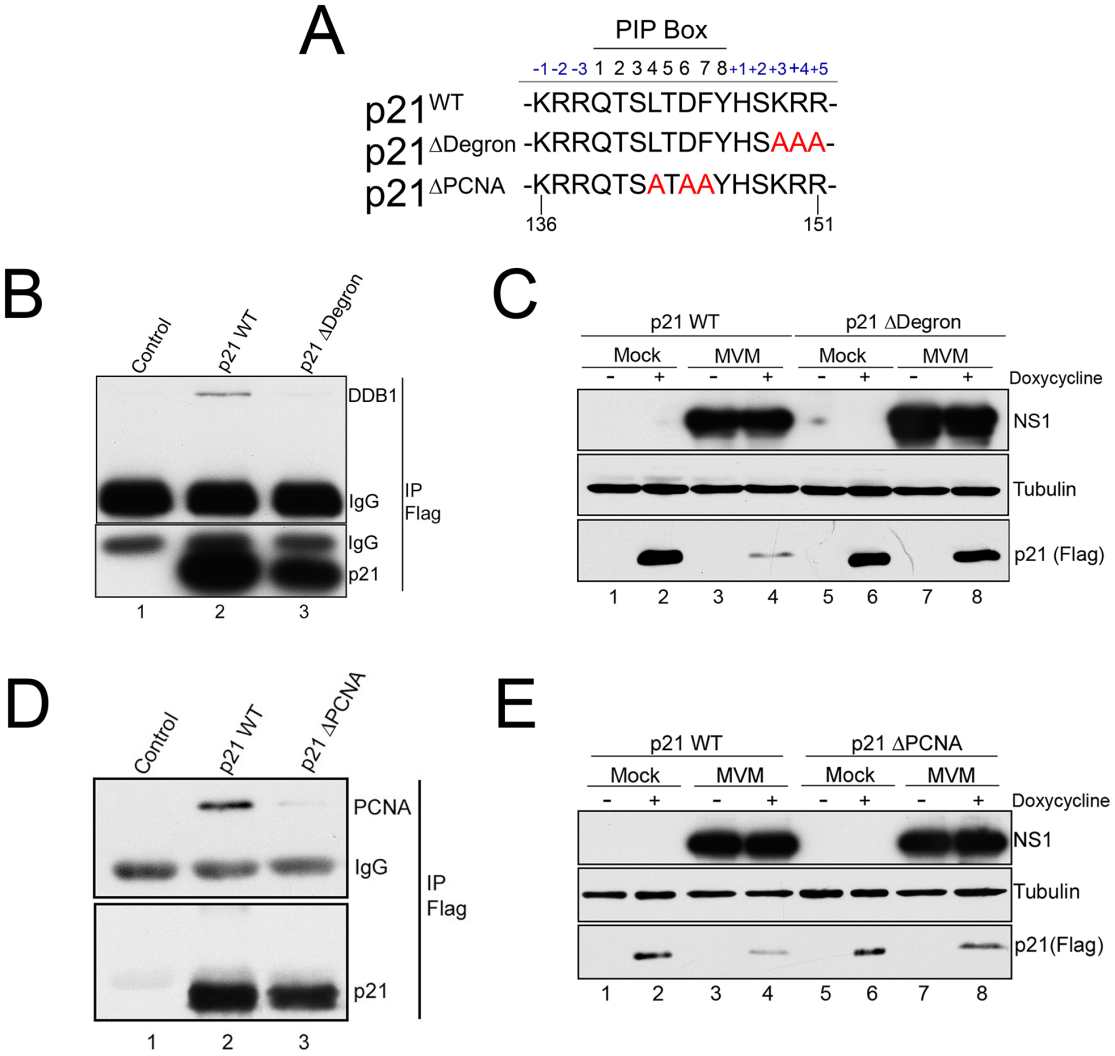
p21 degradation during MVM requires interaction with PCNA and the CRL4^Cdt2^ ligase complex. **A**) Illustration of the p21 PIP/degron region (amino acid 136 to 151) of wild-type murine p21 (p21^WT^), p21^Δdegron^, and p21^ΔPIP^. Mutations are shown in red. **B**) *p21^Δdegron^ does not interact with the CRL4^Cdt2^ complex*. 293T were cells transfected with constructs expressing FLAG-tagged p21^WT^ (lane 2), p21^Δdegron^ (lane 3) or control plasmid (lane 1). Cells were harvested at 48 hr. Lysates were immunoprecipitated using with FLAG antibody and blotted against the indicated proteins. **C**) *MVM degradation of p21 requires its interaction with the CRL4^Cdt2^ ligase complex*. Murine A9 cell lines stably expressing p21^WT^ and p21^Δdegron^ were generated as described. Cells were parasynchronized, released into complete media and mock-infected or infected with MVM at an MOI of 10. At 20 hr pi cells were treated with doxycycline to induce p21 expression. Cells were harvested 6 hrs later and processed for western blotting using the indicated antibodies. **D**) *p21^ΔPCNA^ does not interact with PCNA*. Experiment performed as for Figure 4B. **E**) *MVM degradation of p21 requires its interaction with PCNA*. Experiment performed as for 4C.

To investigate the importance of these interactions for the MVM-dependent targeting of p21 by the CRL4^Cdt2^ ligase we generated stable murine cell lines *via* lentivirus transduction that conditionally expressed FLAG-tagged wild-type or mutant p21 (p21^WT^, p21^ΔDegron^, p21^ΔPCNA^, mutations shown in Figure 4A) in a doxycycline-responsive manner. As expected, MVM infection of a p21^WT^ expressing cell line resulted in degradation of the tagged p21 (Figure 4C and 4E, compare lanes 2 to 4), which could be prevented by treating cells with the proteasome inhibitor MG132 (Figure S4, panel A), or *via* siRNA knockdown of CRL4^Cdt2^ components (Figure S4, panels B and C). These results suggested that the depletion of p21 in these cell lines occurred *via* a similar mechanism to that of the endogenous p21. The lysine at the p21 +4 position, 3′ to its PIP box, has been reported to be required for interaction of p21 with the CRL4^Cdt2^ ligase complex *via* Cdt2. Mutation of the +3 to +5 amino acids KRR to AAA (p21^Δdegron^) abolished p21 interaction with the CRL4^Cdt2^ complex following its transient transfection as reflected by loss of interaction with DDB1 (Figure 4B, compare lanes 2 to 3). Murine cell lines that expressed the p21^Δdegron^ mutant were generated, and when infected with MVM, in contrast to cell lines expressing wild-type p21 (p21^WT^), the p21^Δdegron^ protein was resistant to degradation (Figure 4C, compare lanes 6 and 8 to lanes 2 and 4). This suggested that MVM-induced p21 degradation required interaction of p21 with the CRL4^Cdt2^ ubiquitin ligase complex.

The CRL4^Cdt2^ ligase also requires PCNA as a cofactor for targeting of its substrates [13]. Mutation of the p21 PIP box (p21^ΔPCNA^, Figure 4A) abrogated interaction with PCNA (Figure 4D, compare lane 3 to 2), and the p21^ΔPCNA^ protein expressed in murine cells was not degraded as efficiently as the wild-type protein (p21^WT^) following infection (Figure 4E, compare lanes 8 and 6 to 4 and 2). These results suggested that PCNA-binding was also essential for effective CRL4^Cdt2^ targeting of p21 during MVM infection.

### Stable p21 that retains interaction with PCNA inhibits MVM replication

p21 interaction with PCNA has been shown to interfere with DNA polymerase δ−mediated cellular DNA replication [5], [6]. PCNA is an important cofactor for MVM replication; it contributes to MVM replication *in vitro* [8], and is recruited to APAR bodies during infection. Thus, we investigated whether MVM-dependent depletion of p21 during infection, mediated by the CRL4^Cdt2^ E3 ubiquitin ligase, promoted efficient replication of the MVM genome by abrogating its inhibition of PCNA.

**Figure 5:**
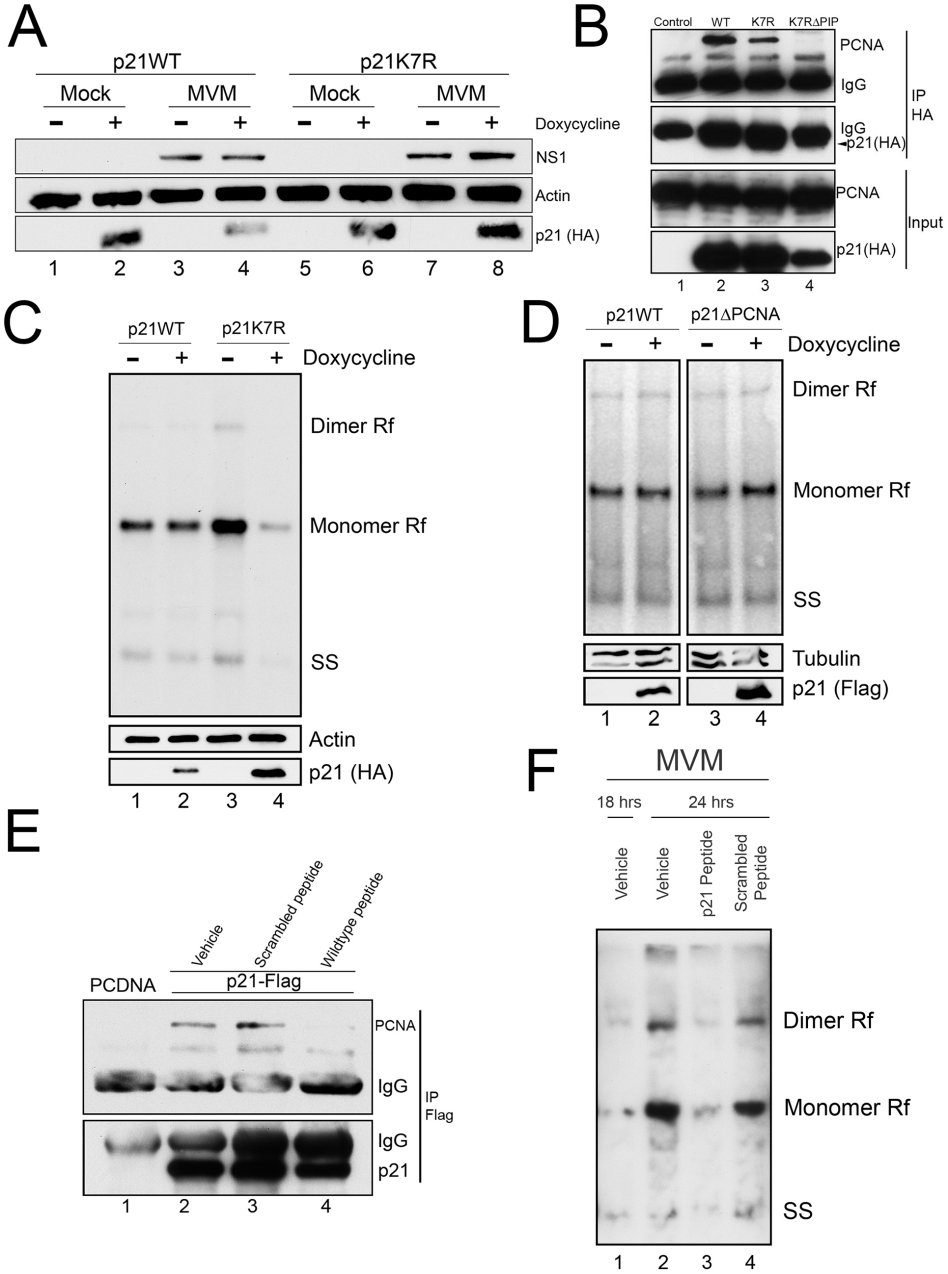
A stabilized p21 that binds to PCNA inhibits MVM replication. **A**) *p21 degradation by MVM requires a ubiquitin-modifiable lysine*. Murine A9 cell lines stably expressing HA-tagged wild-type p21 (p21^WT^) or a mutant in which all seven lysines have been mutated to arginines (p21^K7R^) were generated. p21 degradation assay was performed as described in 3C. **B**) *p21^K7R^ retains interaction with PCNA*. Control plasmid (lane 1), p21^WT^ (lane 2), p21^K7R^ (lane 3), and p21^K7R^ with additional mutations that disrupt PCNA interaction (p21^K7RΔPIP^, lane 4) were transfected into 293T cells. At 48 hr cells were lysed, immunoprecipitated with HA antibodies and blotted using the indicated antibodies. The p21^K7RΔPIP^ was expressed at lower levels in this experiment however interaction with PCNA was not detected even upon longer exposure. **C**) *p21^K7R^ inhibits MVM replication*. p21^WT^ and p21^K7R^ cell lines were parasynchronized, released and infected with MVM at an MOI of 0.5. At 16 hr pi cells were treated with doxycycline to induce p21 expression and harvested 8 hrs later. Cells were processed for Southern blotting (top panel), or for western blotting using the indicated antibodies (bottom panels). A representative experiment is shown; quantifications in the text reflect three independent experiments. **D**) *p21^ΔPCNA^ does not inhibit MVM replication*. p21^WT^ and p21^ΔPCNA^ cells were treated, processed and standardized as in 4C. **E**) *A p21 peptide competitively binds to PCNA*. 293T cells were transfected with a construct expressing FLAG-tagged p21 (lanes 2 to 4) or control plasmid (PCDNA, lane 1) and processed for co-immunoprecipitation as in 4D. Treatment with wild-type but not scrambled peptide reduced interaction of p21 with PCNA. **F**) *A p21-derived peptide inhibits MVM replication*. Murine A9 cells were parasynchronized, released and infected with MVM at an MOI of 1. At 18 hr pi control cells were harvested (lane 1) and remainder were treated with vehicle (lane 2), wild-type peptide (lane 3) or scrambled peptide (lane 4) for 6 hrs. At 24 hr pi cells were harvested and processed for Southern blotting. A representative experiment is shown; the experiment was done three times.

Unexpectedly, the p21^ΔDegron^ mutant, although stable, interacted poorly with PCNA for reasons not yet clear (data not shown). As a result, we could not use cell lines expressing this mutant to determine whether stabilized, p21 affected MVM replication *via* PCNA binding. Thus we generated a murine cell line conditionally expressing a mutant p21 in which all seven lysines in p21 were changed to arginine [p21^K7R^, a similar mutation has been reported by others [23]]. Similar to the p21^ΔDegron^ mutant, the p21^K7R^ protein was resistant to degradation following MVM infection (Figure 5A, compare lanes 6 and 8 to 2 and 4), yet retained substantial interaction with PCNA in transient transfection assays (Figure 5B, compare lane 3 to lanes 2 and 4). Whereas induction of p21^WT^ expression for 8 hrs after infection had little effect on MVM replication (Figure 5C, compare lanes 1 and 2), p21^K7R^ expression reduced replication by up to 3 fold (Figure 5C, compare lanes 3 and 4). Importantly, in contrast, the p21^ΔPCNA^ mutant-expressing cell line, which expressed a stable p21 which no longer could interact with PCNA (Figure 4D and 4E), failed to inhibit MVM replication upon induction (Figure 5D, compare lanes 3 and 4). This was also the case with the p21^ΔDegron^ mutant-expressing cell lines (data not shown). Furthermore, cell lines expressing a mutant of p21^K7R^ in the PIP box-mutated background (p21^K7RΔPIP^) (see Figure 5B, lane 4) also failed to inhibit MVM replication (Figure S5), demonstrating that absent PCNA binding, the K7R mutation itself had no deleterious effect on MVM replication. All the mutants tested were recruited to APAR bodies during MVM infection (Figure S6).

To confirm that the p21-PCNA interaction mediated the inhibitory role of p21 during infection, we made use of a previously described peptide containing 20 residues derived from sequences comprising the p21 PIP box [24] fused to a 16-mer penetratin motif to facilitate cellular entry [25]. A scrambled version of the p21 peptide linked to the penetratin peptide was used as control. Following application to murine cells, the wild-type (Figure 5E, lane 4), but not the scrambled version (Figure 5E, lane 3), disrupted the interaction between over-expressed FLAG-tagged p21 and endogenous PCNA, demonstrating that the peptide could competitively interact with PCNA. Subsequently, while treatment of cells with the scrambled peptide had no effect on MVM replication (Figure 5F, compare lanes 2 and 4), treatment of cells with the wild-type p21 PCNA-binding peptide significantly inhibited MVM replication (Figure 5F, compare lanes 2 and 3). These results indicated that a stabilized p21 interaction with PCNA was detrimental to viral replication. Thus, while interaction with PCNA was important for targeting p21 to the re-localized CRL4^Cdt2^ ligase, efficient viral replication required subsequent depletion of p21 and consequent abrogation of its inhibition of PCNA.

## Discussion

Parvoviruses and other small DNA viruses rely on host polymerases to replicate their genomes. How the replication machinery is exploited to sustain parvovirus replication in G2-arrested cells, which normally contain potentially inhibitory cellular proteins such as p21, is not fully understood. p21 levels are high in G1, low in S-phase, restored in G2 phase, and are regulated by proteasomal degradation during cell cycle progression [4]. We and others have previously reported that expression of the parvoviral NS1 protein leads to increases in p21 levels [2], [3]. Additionally, p53, a transcriptional activator of p21, is significantly up-regulated and activated throughout MVM infection. Remarkably however, while these signals lead to increased p21 RNA accumulation, p21 protein levels remain low throughout infection [2]. Here we have identified the mechanism by which p21 was degraded upon parvovirus infection, and identified the consequence of this for virus replication.

Degradation of p21 during S-phase and in response to DNA damaging agents such as UV treatment is programmed by the CRL4^Cdt2^ ubiquitin ligase [13] and in this manuscript we have demonstrated that the same ligase targets p21 for degradation during MVM infection. The circumstances surrounding p21 degradation and the signals leading to it in the context of parvoviral infection, however, differ from how it occurs during S-phase. During MVM infection p21 loss persists for extended periods of time while virus is replicating in G2 arrested cells in the presence of high amounts of activated p53 and NS1 [2]. Additionally, whereas ATR activity is important for p21 degradation in response to various DDR-inducing agents [23], [26], the ATR substrate Chk1 is not activated during MVM replication (Adeyemi and Pintel, in preparation), suggesting that p21 degradation during infection may occur independently of ATR activity.

During MVM infection the CRL4^Cdt2^ ligase is recruited to viral replication centers. Recently, the CRL4^Cdt2^ ligase was shown to be recruited to cellular chromatin *via* direct PCNA interaction [21]. It is not yet clear whether similar mechanisms mediate CRL4^Cdt2^ recruitment to MVM APAR bodies; however, it appears that PCNA recruitment to MVM chromatin may represent a critical step leading to viral hijacking of the CRL4^Cdt2^ ligase. Neither interaction with PCNA nor the ligase was required for recruitment of p21 to replication centers, as all the mutants tested were relocalized to APAR bodies.

Stabilization of p21 *via* CRL4^Cdt2^ depletion led to reduced MVM replication subsequent to the initiation of genome replication following S-phase entry. This is the first published demonstration of the requirement and re-localization to replication centers of a specific cellular ubiquitin ligase during autonomous parvovirus replication. p21 is a potent inhibitor of CDKs and PCNA. Exactly how stabilized p21 inhibited MVM replication is not fully clear; however, interaction with PCNA mediated its inhibitory role. Using inducible cell lines expressing wild-type and mutant p21 proteins we demonstrated that p21 degradation during infection required motifs that mediate interaction with both Cdt2 and PCNA. PCNA is recruited to MVM chromatin [18] and is essential for parvovirus replication [27], [28]. Overexpression of a stable mutant p21 that retained interaction with PCNA, but could not be targeted for degradation by the CRL4^Cdt2^ ligase, led to reduced virus replication. Stable p21 mutants unable to bind PCNA did not affect MVM replication, indicating that other potential inhibitory functions of p21, such as CDK binding and promoter repression, were not detrimental to viral replication absent PCNA interaction. Additionally, introduction of a p21-derived peptide which maintained PCNA interaction abolished viral replication. Thus, p21 interaction with PCNA (and Cdt2) was necessary for targeting of p21 to the co-localized CRL4^Cdt2^ ligase, and its subsequent depletion also prevented its sustained interaction with PCNA that would otherwise be inhibitory to viral replication. Although our earlier work had suggested that Cdk2 activity was required for MVM replication and that p21 degradation might be necessary to prevent inhibition of CDKs [2], we have recently shown that Chk2 activation during MVM infection results in CDC25A degradation leading to partial CDK2 inhibition, independent of p21 [29]. Thus, while some level of CDK activity is required for MVM replication, prevention of CDK inhibition is not likely to be the critical reason for p21 degradation.

p21 has been shown to inhibit MVM replication *in vitro*, and this effect was shown to be overcome by the addition of increasing amounts of PCNA [8]. p21 binds to PCNA *via* its PIP box, a conserved motif shared by substrates of the CRL4^Cdt2^ ligase, as well as cellular proteins such as DNA polymerase δ, that are essential for replication but escape ubiquitin targeting due to the absence of a PIP degron [13]. The p68 subunit of DNA polymerase δ binds to PCNA *via* a similar hydrophobic pocket recognized by the p21 PIP box. Thus, while the mechanism of p21 inhibition of the DNA polymerase δ/PCNA complex has not been clearly resolved, a stabilized high-affinity interaction of p21 with the DNA polymerase δ binding pocket within PCNA could directly inhibit viral replication by competing with DNA polymerase δ for binding to its cofactor [30].

Due to its myriad effects on cell cycle progression and cancer, several viruses make use of different strategies to inactivate p21 during infection. For example, several oncogenic DNA viruses indirectly inhibit p21 by targeting p53 [31]. Additionally, papillomaviruses HPV E7 proteins sequester p21 thereby preventing its interaction with PCNA and CDKs [32], [33]. KSHV encodes a microRNA that down-regulates p21 in order to prevent cell cycle arrest [34]. p21 restricts HIV in myeloid cells of certain patients [35], [36], and a recent report demonstrated that this restriction may occur *via* ribonucleotide reductase-2 repression resulting in inhibition of dNTP biosynthesis [37]. Thus, cellular components required for viral genomic replication, such as dNTPs and polymerase cofactors, may be critically dependent on p21 depletion. Our findings present first a novel mechanism of p21 antagonism by a virus, namely, the use of a cellular E3 ligase recruited to viral replication factories. Further, we shown that efficient virus replication depends on the depletion of p21 to prevent its inhibition of PCNA. PCNA is an important cofactor for the replication of several DNA viruses. PCNA-mediated degradation of p21 in the context of viral infection may emerge as an important paradigm for allowing sustained viral replication of DNA polymerase-δ dependent viruses in infected cells.

## Materials and Methods

### Cell lines and drug treatments

Murine A9 and Human 293T cell lines were propagated as previously described [38], [39]. Stable doxycycline-inducible A9 cell lines were generated by infection of A9 cells with pseudotyped virus using the pINDUCER20 lentiviral system [40]. Cell lines were selected with 800 µg/ml of geneticin (Gibco) and maintained like regular A9s except for addition of geneticin. A9 cells were parasynchronized in G0 by isoleucine deprivation as previously described [1]. pINDUCER20 lentiviral transformed cell lines were induced with 500 ng/mL doxycycline hydrochloride (MP Biomedical). MG132 (Calbiochem) was added at a final concentration of 10 µM.

### Viruses and infections

Wild-type MVMp was propagated and titered in murine A9 cell lines as described [1]. Pseudotyped viruses were generated by co-transfecting equal concentrations of HIV Gag/Pol, VSV-G and pINDUCER20 plasmids into 293T cells. Supernatants were collected and used to infect A9 cells.

### Plasmids and DNA transfection

Murine wild-type p21 cDNA (Origene) was cloned into p3XFLAG-CMV 7.1 (Sigma). Additionally, p21 was tagged with a 3× HA tag by PCR mutagenesis. p21^K7R^ was generated by PCR mutagenesis. p21^ΔPCNA^, p21^Δdegron^ and p21^K7RΔPIP^ (Q139A, L142A, F145A, Y146A) mutants were generated by site-directed mutagenesis (Agilent). FLAG and HA-tagged wild-type and mutant p21 were cloned into pDONR221 (Invitrogen) and pINDUCER20 using BP and LR clonase kits (Invitrogen) respectively. pINDUCER reagents were a gift from Guang Hu at NIH/NIEHS. DNA transfection was performed using LipoD293 (Signagen) or Lipofectamine (Invitrogen).

### RNA interference

A9 cells were transfected twice with 40 nM Control siRNA (Qiagen #1022076), Cdt2 (DTL) siRNA (Dharmacon #L-045921-01-0005), Cul4A (Dharmacon #L-052208-00-0005) or DDB1 siRNA (Dharmacon #L-043146-01-0005). siRNA transfections were performed using HiPerfect (Qiagen).

### Peptides

Wild-type p21 (KRRQTSMTDFYHSKRRLIFSRQIKIWFQNRRMKWKK) and scrambled p21 (KSTARHTKLSAQRYIRSFARRQIKIWFQNRRMKWKK) were purchased from Peptide 2.0 Inc. The peptides consist of p21-derived or scrambled sequences fused to penetratin – a 16 amino acid nuclear internalization sequence derived from the Antennapedia homeodomain [25]. Peptides were added to cells at 25 µM.

### Antibodies

Commercially available antibodies used in this study were obtained from Bethyl (Cdc20, cat# A301-180A), Cell Signaling (Set8, cat# 2996S), Invitrogen (DDB1, cat# 399901; p21, cat# 556430), Millipore (Cdt1, cat# 06-1295; PCNA, cat# CBL407), Novus (Cullin 4A, cat# NB100-2267), Pierce (Actin, cat# MA515739), Sigma (FLAG, cat# F1804; HA, cat# H9658; Tubulin, cat# T4026), and Upstate (Cyclin A, cat# 06-138). Cdt2 antibody was a generous gift from Anindya Dutta (University of Virginia). All secondary antibodies were from Invitrogen. NS1 (CE10) and NS1/2 (M55) were previously described [1].

### Immunoblot analysis

Immunoblots were performed as described previously [1]. Protein concentrations were quantified by Bradford assay and equal amounts of lysates were run.

### Co-immunoprecipitation

FLAG and HA-tagged p21 was immunoprecipitated from 293T cells as previously described [12]. FLAG beads (Sigma) and HA antibodies were used for IP.

### Immunofluorescence (IF)

For IF, A9 cells were grown on glass coverslips in 35 mm dishes and infected with MVMp at an MOI of 10. At 24 hr pi, cells were washed twice with cold phosphate-buffered saline (PBS) solution and then with cytoskeleton buffer [10 mM piperazine-N,N′-bis(2-ethanesulfonic acid) (PIPES), pH 6.8, 100 mM NaCl, 300 mM sucrose, 1 mM MgCl_2_, 1 mM EGTA]. Afterwards cells were pre-extracted for in cytoskeletal buffer containing 0.5% Triton X-100, protease and phosphatase inhibitors for 3 minutes on ice, washed, fixed with 4% paraformaldehyde and stained for the indicated proteins. Nuclei were visualized by staining with To-Pro-3 (Invitrogen). The coverslips were mounted in Fluoromount-G (Southern Biotech) and images were acquired using a Zeiss LSM 510 META confocal microscope. All images were captured using an objective of 63×.

### Analysis of viral DNA

Southern blots were carried out as previously described [38], using whole MVM genome probes. Loading of DNA samples was normalized using a nanodrop spectrophotometer. This procedure was verified using probes on Southern blots against mitochondrial DNA. Unless otherwise indicated, infections were carried out at a low MOI in order to maximize effects of siRNA knockdowns and overexpression analyses on viral replication. Representative Southern Blots are shown; quantifications in the text reflect multiple knockdown experiments.

### Cell cycle analysis

A9 cells were fixed in 70% ethanol overnight at 4°C. Cells were then pelleted, washed in PBS and resuspended in PBS containing 0.2 mg/ml RNAse A for 1 hr at 37°C, then propidium iodide was added at 40 µg/ml. Flow cytometry was performed using FACScan (BD biosciences). Data were analyzed using Summit software (Beckman Coulter).

## Supporting Information

Figure S1 **Set8 but not Cdt1 is degraded during MVM infection.** Parasynchronous A9 cells were infected with MVM at an MOI of 10. Western blots were performed against the indicated proteins. MVM infection resulted in reduction in Set8 protein levels. Cdt1, whose expression levels were not altered, appeared to run as a doublet upon MVM infection.
(TIF)

Figure S2 **Cdt2 knockdown does not significantly affect S-phase entry.** **A**) Schematic illustrating the experimental protocol for S1B. **B**) Murine A9 cells were treated with siRNA as shown in S1A and were processed for flow cytometry as described in Experimental Procedures. Mock T0 cells were processed at time of release from parasynchronization and show a majority of cells in G0/G1. siRNA-transfected T16 cells were processed at 16 hr post-release and showed the expected reduction in G0/G1 levels compared to mock T0 cells. There was no significant reduction in S-phase accumulation upon Cdt2 knockdown compared to control siRNA treatment; however, the G2/M to S ratio under these conditions varies between experiments. PI stands for propidium iodide.
(TIF)

Figure S3 **APC/C E3 ubiquitin ligase is not recruited to APAR bodies.** Murine A9 cells were mock infected or infected with MVM at an MOI of 10. At 32 hr pi cells were processed for immunofluorescence as described in experimental procedures, without detergent pre-extraction, using antibodies against NS1 and Cdc20.
(TIF)

Figure S4 **Overexpressed p21 is degraded in a proteasome and CRL4^Cdt2^ -dependent manner following MVM infection.** **A**) Parasynchronized murine A9 cell lines stably expressing FLAG-tagged p21^WT^ were mock infected or infected with MVM at an MOI of 10. At 18 hr pi cells were treated with doxycycline to induce p21 expression and treated with MG132 as indicated. Cells were harvested 6 hrs later and processed for western blotting using the antibodies indicated. **B and C**) p21^WT^ cell lines were treated with control siRNA or siRNA targeted to Cul4A (B) or DDB1 (C), as indicated, during parasynchronization. Cells were released and mock infected or infected with MVM at an MOI of 10. At 18 hr pi cells were treated with doxycycline to induce p21 expression. Cells were harvested at 24 hr pi and processed for western blotting using the antibodies indicated.
(TIF)

Figure S5 **p21^K7RΔPIP^ does not inhibit MVM replication.** p21^WT^ and p21^K7RΔPIP^ cell lines were parasynchronized, released and infected with MVM at an MOI of 0.5. At 16 hr pi cells were treated with doxycycline to induce p21 expression and harvested 8 hrs later. Cells were processed for Southern blotting (top panel), or for western blotting using the indicated antibodies (bottom panels).
(TIF)

Figure S6 **p21 mutants are recruited to MVM replication compartments.** Murine A9 cell lines stably expressing FLAG-tagged p21^PCNA^, p21^ΔDegron^ or HA-tagged p21^K7R^ or p21^K7RΔPIP^ were mock infected or infected with MVM at an MOI of 10. At 18 hr pi cells were treated with doxycycline to induce p21 expression. At 24 hr pi cells were processed for IF using antibodies against NS1 and FLAG or HA.
(TIF)

## References

[1] Adeyemi RO, Landry S, Davis ME, Weitzman MD, Pintel DJ (2010) Parvovirus minute virus of mice induces a DNA damage response that facilitates viral replication. PLoS Pathog 6: e1001141. 20949077 10.1371/journal.ppat.1001141 PMC2951379

[2] Adeyemi RO, Pintel DJ (2012) Replication of minute virus of mice in murine cells is facilitated by virally induced depletion of p21. J Virol 86: 8328–8332. 22623787 10.1128/JVI.00820-12 PMC3421664

[3] Op De Beeck A, Sobczak-Thepot J, Sirma H, Bourgain F, Brechot C, et al (2001) NS1- and minute virus of mice-induced cell cycle arrest: involvement of p53 and p21(cip1). J Virol 75: 11071–11078. 11602746 10.1128/JVI.75.22.11071-11078.2001 PMC114686

[4] Abbas T, Dutta A (2009) p21 in cancer: intricate networks and multiple activities. Nat Rev Cancer 9: 400–414. 19440234 10.1038/nrc2657 PMC2722839

[5] Chen J, Jackson PK, Kirschner MW, Dutta A (1995) Separate domains of p21 involved in the inhibition of Cdk kinase and PCNA. Nature 374: 386–388. 7885482 10.1038/374386a0

[6] Chen J, Peters R, Saha P, Lee P, Theodoras A, et al (1996) A 39 amino acid fragment of the cell cycle regulator p21 is sufficient to bind PCNA and partially inhibit DNA replication in vivo. Nucleic Acids Res 24: 1727–1733. 8649992 10.1093/nar/24.9.1727 PMC145832

[7] Waga S, Hannon GJ, Beach D, Stillman B (1994) The p21 inhibitor of cyclin-dependent kinases controls DNA replication by interaction with PCNA. Nature 369: 574–578. 7911228 10.1038/369574a0

[8] Bashir T, Horlein R, Rommelaere J, Willwand K (2000) Cyclin A activates the DNA polymerase delta -dependent elongation machinery in vitro: A parvovirus DNA replication model. Proc Natl Acad Sci U S A 97: 5522–5527. 10792046 10.1073/pnas.090485297 PMC25861

[9] Randow F, Lehner PJ (2009) Viral avoidance and exploitation of the ubiquitin system. Nat Cell Biol 11: 527–534. 19404332 10.1038/ncb0509-527

[10] Abbas T, Sivaprasad U, Terai K, Amador V, Pagano M, et al (2008) PCNA-dependent regulation of p21 ubiquitylation and degradation via the CRL4Cdt2 ubiquitin ligase complex. Genes Dev 22: 2496–2506. 18794347 10.1101/gad.1676108 PMC2546691

[11] Kim Y, Starostina NG, Kipreos ET (2008) The CRL4Cdt2 ubiquitin ligase targets the degradation of p21Cip1 to control replication licensing. Genes Dev 22: 2507–2519. 18794348 10.1101/gad.1703708 PMC2546690

[12] Nishitani H, Shiomi Y, Iida H, Michishita M, Takami T, et al (2008) CDK inhibitor p21 is degraded by a proliferating cell nuclear antigen-coupled Cul4-DDB1Cdt2 pathway during S phase and after UV irradiation. J Biol Chem 283: 29045–29052. 18703516 10.1074/jbc.M806045200 PMC2662008

[13] Havens CG, Walter JC (2011) Mechanism of CRL4(Cdt2), a PCNA-dependent E3 ubiquitin ligase. Genes Dev 25: 1568–1582. 21828267 10.1101/gad.2068611 PMC3182024

[14] Soria G, Gottifredi V (2010) PCNA-coupled p21 degradation after DNA damage: The exception that confirms the rule? DNA Repair (Amst) 9: 358–364. 20060369 10.1016/j.dnarep.2009.12.003 PMC2915755

[15] Abbas T, Shibata E, Park J, Jha S, Karnani N, et al (2010) CRL4(Cdt2) regulates cell proliferation and histone gene expression by targeting PR-Set7/Set8 for degradation. Mol Cell 40: 9–21. 20932471 10.1016/j.molcel.2010.09.014 PMC2966975

[16] Centore RC, Havens CG, Manning AL, Li JM, Flynn RL, et al (2010) CRL4(Cdt2)-mediated destruction of the histone methyltransferase Set8 prevents premature chromatin compaction in S phase. Mol Cell 40: 22–33. 20932472 10.1016/j.molcel.2010.09.015 PMC2957874

[17] Jin J, Arias EE, Chen J, Harper JW, Walter JC (2006) A family of diverse Cul4-Ddb1-interacting proteins includes Cdt2, which is required for S phase destruction of the replication factor Cdt1. Mol Cell 23: 709–721. 16949367 10.1016/j.molcel.2006.08.010

[18] Bashir T, Rommelaere J, Cziepluch C (2001) In vivo accumulation of cyclin A and cellular replication factors in autonomous parvovirus minute virus of mice-associated replication bodies. J Virol 75: 4394–4398. 11287588 10.1128/JVI.75.9.4394-4398.2001 PMC114184

[19] Cziepluch C, Lampel S, Grewenig A, Grund C, Lichter P, et al (2000) H-1 parvovirus-associated replication bodies: a distinct virus-induced nuclear structure. J Virol 74: 4807–4815. 10775619 10.1128/jvi.74.10.4807-4815.2000 PMC112003

[20] Amador V, Ge S, Santamaria PG, Guardavaccaro D, Pagano M (2007) APC/C(Cdc20) controls the ubiquitin-mediated degradation of p21 in prometaphase. Mol Cell 27: 462–473. 17679094 10.1016/j.molcel.2007.06.013 PMC2000825

[21] Havens CG, Shobnam N, Guarino E, Centore RC, Zou L, et al (2012) Direct role for proliferating cell nuclear antigen in substrate recognition by the E3 ubiquitin ligase CRL4Cdt2. J Biol Chem 287: 11410–11421. 22303007 10.1074/jbc.M111.337683 PMC3322809

[22] Havens CG, Walter JC (2009) Docking of a specialized PIP Box onto chromatin-bound PCNA creates a degron for the ubiquitin ligase CRL4Cdt2. Mol Cell 35: 93–104. 19595719 10.1016/j.molcel.2009.05.012 PMC2744448

[23] Bendjennat M, Boulaire J, Jascur T, Brickner H, Barbier V, et al (2003) UV irradiation triggers ubiquitin-dependent degradation of p21(WAF1) to promote DNA repair. Cell 114: 599–610. 13678583 10.1016/j.cell.2003.08.001

[24] Warbrick E, Lane DP, Glover DM, Cox LS (1995) A small peptide inhibitor of DNA replication defines the site of interaction between the cyclin-dependent kinase inhibitor p21WAF1 and proliferating cell nuclear antigen. Curr Biol 5: 275–282. 7780738 10.1016/s0960-9822(95)00058-3

[25] Derossi D, Joliot AH, Chassaing G, Prochiantz A (1994) The third helix of the Antennapedia homeodomain translocates through biological membranes. J Biol Chem 269: 10444–10450. 8144628

[26] Lee JY, Yu SJ, Park YG, Kim J, Sohn J (2007) Glycogen synthase kinase 3beta phosphorylates p21WAF1/CIP1 for proteasomal degradation after UV irradiation. Mol Cell Biol 27: 3187–3198. 17283049 10.1128/MCB.01461-06 PMC1899930

[27] Christensen J, Cotmore SF, Tattersall P (1997) A novel cellular site-specific DNA-binding protein cooperates with the viral NS1 polypeptide to initiate parvovirus DNA replication. J Virol 71: 1405–1416. 8995666 10.1128/jvi.71.2.1405-1416.1997 PMC191197

[28] Christensen J, Tattersall P (2002) Parvovirus initiator protein NS1 and RPA coordinate replication fork progression in a reconstituted DNA replication system. J Virol 76: 6518–6531. 12050365 10.1128/JVI.76.13.6518-6531.2002 PMC136255

[29] Adeyemi RO, Pintel DJ (2014) Parvovirus-induced depletion of cyclin b1 prevents mitotic entry of infected cells. PLoS Pathog 10: e1003891. 24415942 10.1371/journal.ppat.1003891 PMC3887112

[30] Moldovan GL, Pfander B, Jentsch S (2007) PCNA, the maestro of the replication fork. Cell 129: 665–679. 17512402 10.1016/j.cell.2007.05.003

[31] Gartel AL, Radhakrishnan SK (2005) Lost in transcription: p21 repression, mechanisms, and consequences. Cancer Res 65: 3980–3985. 15899785 10.1158/0008-5472.CAN-04-3995

[32] Funk JO, Waga S, Harry JB, Espling E, Stillman B, et al (1997) Inhibition of CDK activity and PCNA-dependent DNA replication by p21 is blocked by interaction with the HPV-16 E7 oncoprotein. Genes Dev 11: 2090–2100. 9284048 10.1101/gad.11.16.2090 PMC316456

[33] Jones DL, Alani RM, Munger K (1997) The human papillomavirus E7 oncoprotein can uncouple cellular differentiation and proliferation in human keratinocytes by abrogating p21Cip1-mediated inhibition of cdk2. Genes Dev 11: 2101–2111. 9284049 10.1101/gad.11.16.2101 PMC316455

[34] Gottwein E, Cullen BR (2010) A human herpesvirus microRNA inhibits p21 expression and attenuates p21-mediated cell cycle arrest. J Virol 84: 5229–5237. 20219912 10.1128/JVI.00202-10 PMC2863803

[35] Chen H, Li C, Huang J, Cung T, Seiss K, et al (2011) CD4+ T cells from elite controllers resist HIV-1 infection by selective upregulation of p21. J Clin Invest 121: 1549–1560. 21403397 10.1172/JCI44539 PMC3069774

[36] Saez-Cirion A, Hamimi C, Bergamaschi A, David A, Versmisse P, et al (2011) Restriction of HIV-1 replication in macrophages and CD4+ T cells from HIV controllers. Blood 118: 955–964. 21642597 10.1182/blood-2010-12-327106 PMC3148172

[37] Allouch A, David A, Amie SM, Lahouassa H, Chartier L, et al (2013) p21-mediated RNR2 repression restricts HIV-1 replication in macrophages by inhibiting dNTP biosynthesis pathway. Proc Natl Acad Sci U S A 110: E3997–4006 doi: 10.1073/pnas.1306719110 24082141 10.1073/pnas.1306719110 PMC3801060

[38] Choi EY, Newman AE, Burger L, Pintel D (2005) Replication of minute virus of mice DNA is critically dependent on accumulated levels of NS2. J Virol 79: 12375–12381. 16160164 10.1128/JVI.79.19.12375-12381.2005 PMC1211553

[39] Nayak R, Pintel DJ (2007) Positive and negative effects of adenovirus type 5 helper functions on adeno-associated virus type 5 (AAV5) protein accumulation govern AAV5 virus production. J Virol 81: 2205–2212. 17166904 10.1128/JVI.02312-06 PMC1865952

[40] Meerbrey KL, Hu G, Kessler JD, Roarty K, Li MZ, et al (2011) The pINDUCER lentiviral toolkit for inducible RNA interference in vitro and in vivo. Proc Natl Acad Sci U S A 108: 3665–3670. 21307310 10.1073/pnas.1019736108 PMC3048138

