# Supplementary figures and images for: Efficient Parvovirus Replication Requires CRL4Cdt2-Targeted Depletion of p21 to Prevent Its Inhibitory Interaction with PCNA
Source: PLoS Pathog. 2014 Apr 3;10(4):e1004055. doi: 10.1371/journal.ppat.1004055 (PMC3974872; doi:10.1371/journal.ppat.1004055)

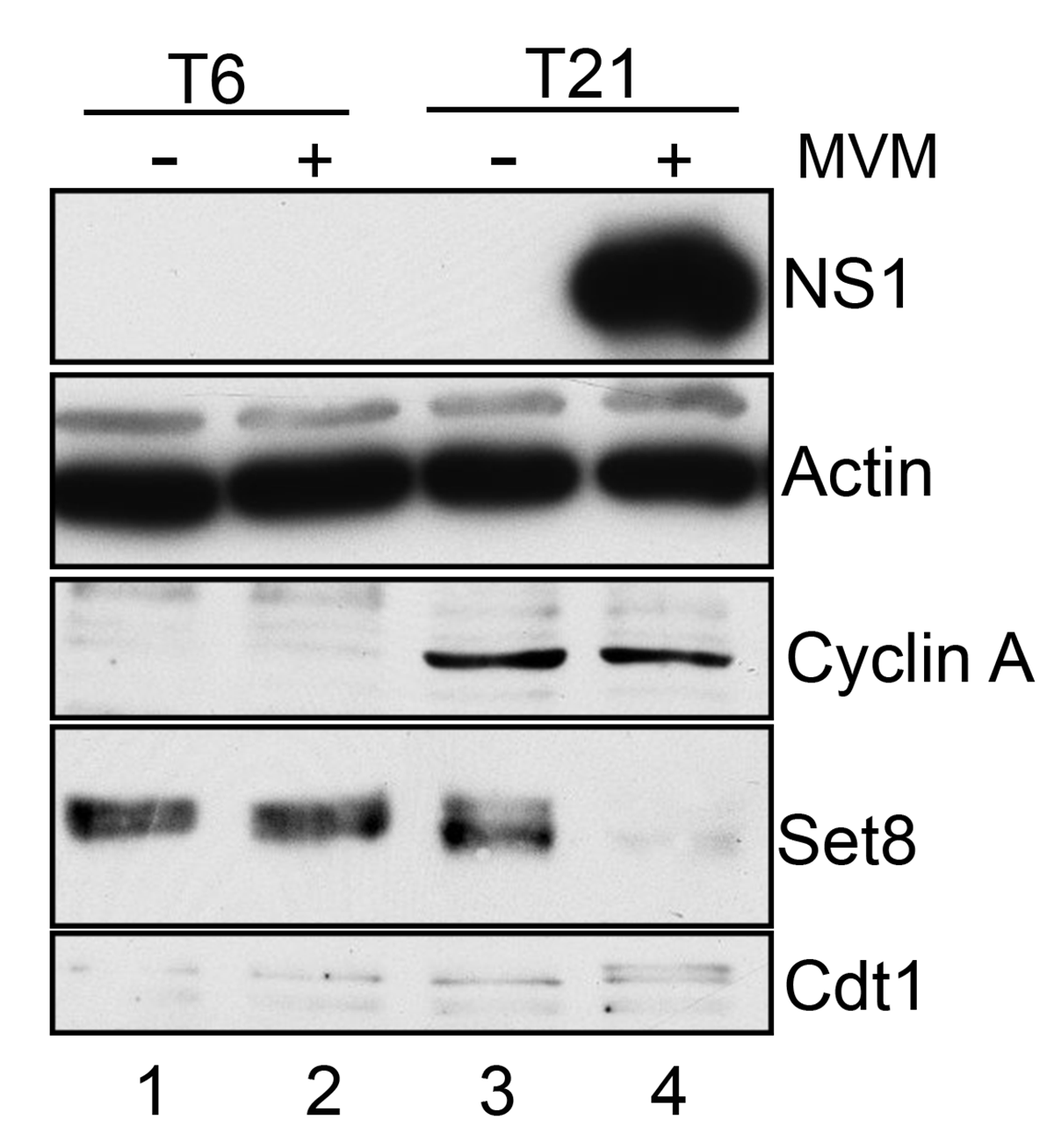

Supplement: Figure S1 — Set8 but not Cdt1 is degraded during MVM infection. Parasynchronous A9 cells were infected with MVM at an MOI of 10. Western blots were performed against the indicated proteins. MVM infection resulted in reduction in Set8 protein levels. Cdt1, whose expression levels were not altered, appeared to run as a doublet upon MVM infection. (TIF) [file ppat.1004055.s001.tif]

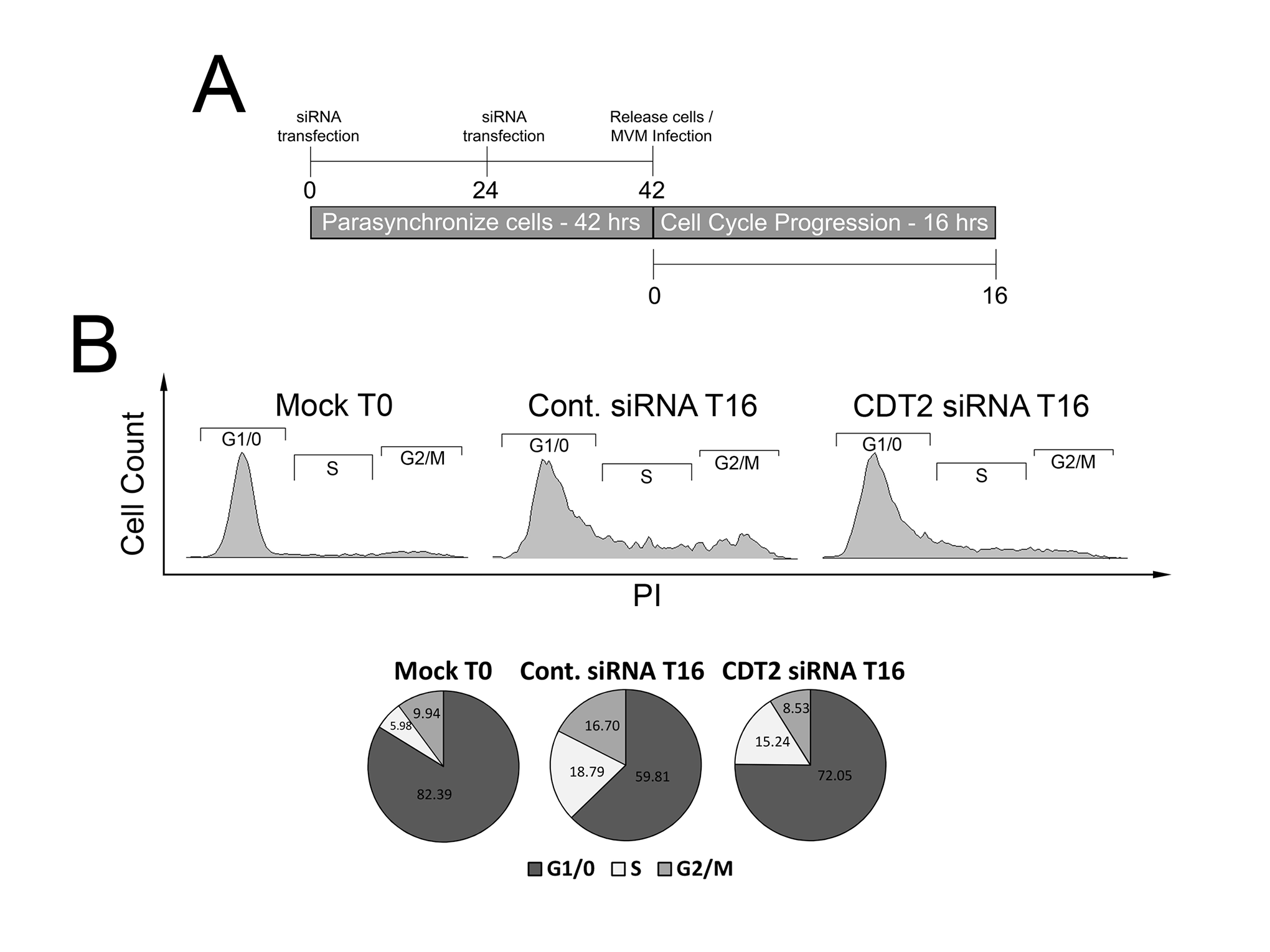

Supplement: Figure S2 — Cdt2 knockdown does not significantly affect S-phase entry. A) Schematic illustrating the experimental protocol for S1B. B) Murine A9 cells were treated with siRNA as shown in S1A and were processed for flow cytometry as described in Experimental Procedures. Mock T0 cells were processed at time of release from parasynchronization and show a majority of cells in G0/G1. siRNA-transfected T16 cells were processed at 16 hr post-release and showed the expected reduction in G0/G1 levels compared to mock T0 cells. There was no significant reduction in S-phase accumulation upon Cdt2 knockdown compared to control siRNA treatment; however, the G2/M to S ratio under these conditions varies between experiments. PI stands for propidium iodide. (TIF) [file ppat.1004055.s002.tif]

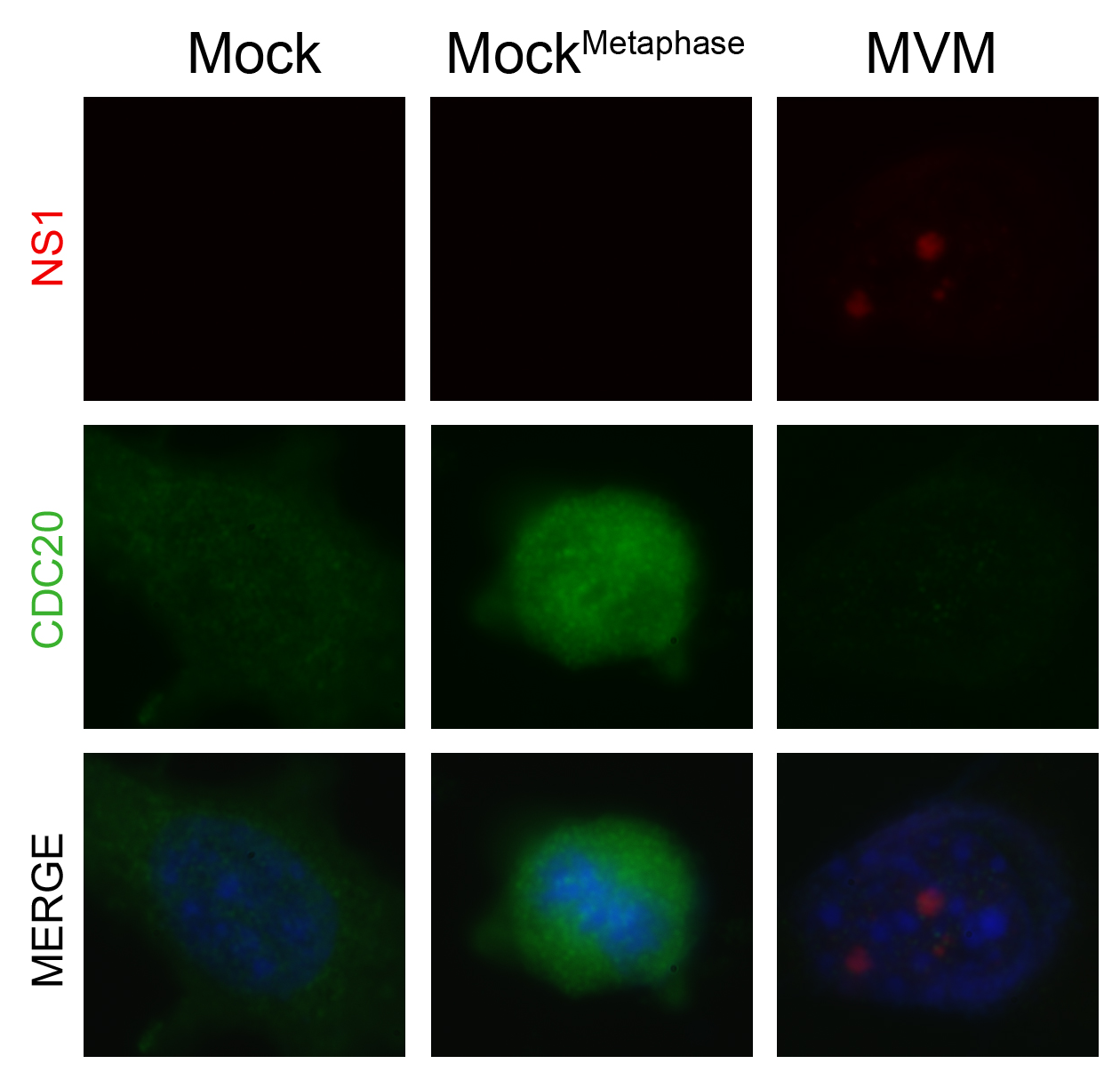

Supplement: Figure S3 — APC/C E3 ubiquitin ligase is not recruited to APAR bodies. Murine A9 cells were mock infected or infected with MVM at an MOI of 10. At 32 hr pi cells were processed for immunofluorescence as described in experimental procedures, without detergent pre-extraction, using antibodies against NS1 and Cdc20. (TIF) [file ppat.1004055.s003.tif]

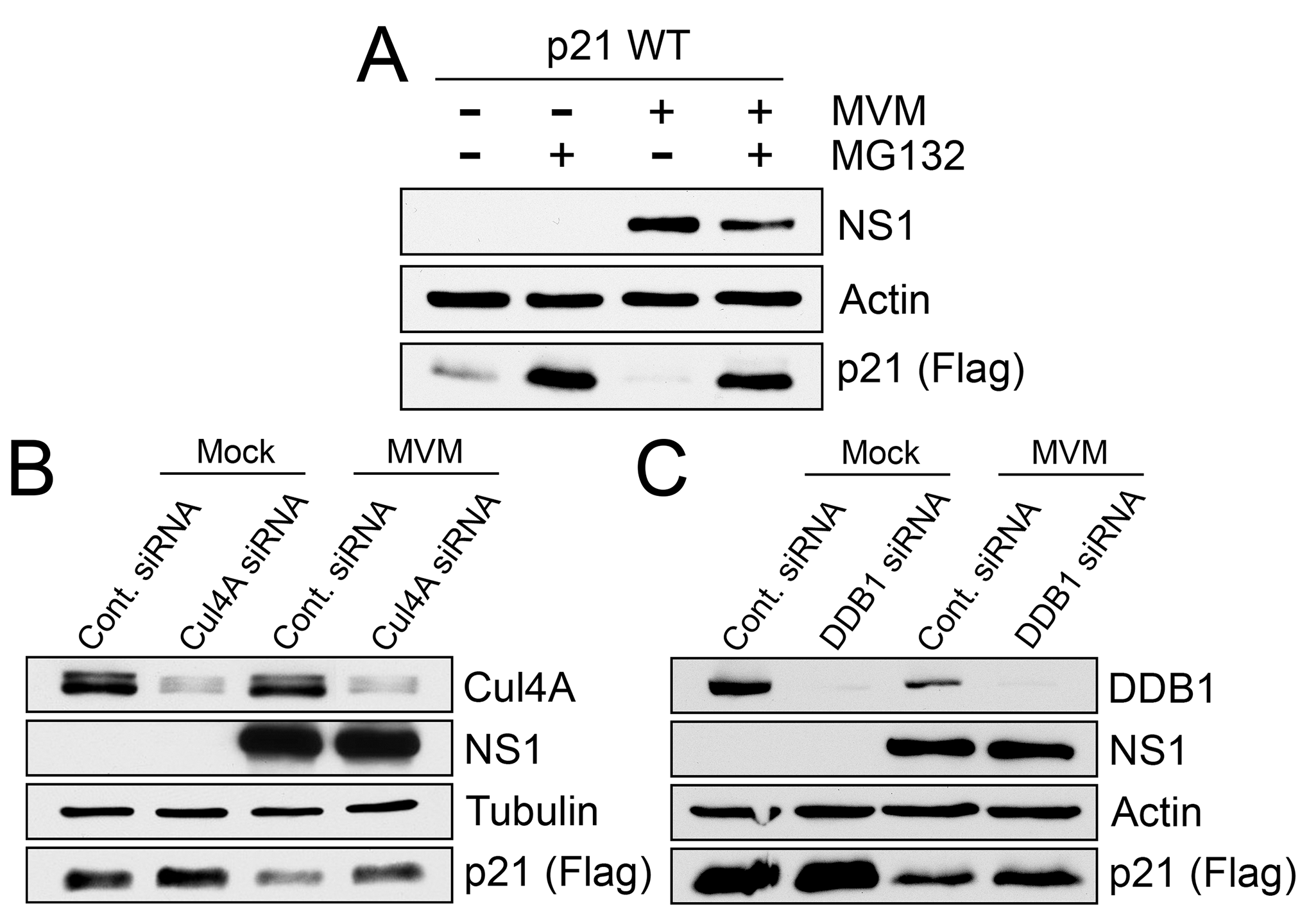

Supplement: Figure S4 — Overexpressed p21 is degraded in a proteasome and CRL4Cdt2 -dependent manner following MVM infection. A) Parasynchronized murine A9 cell lines stably expressing FLAG-tagged p21WT were mock infected or infected with MVM at an MOI of 10. At 18 hr pi cells were treated with doxycycline to induce p21 expression and treated with MG132 as indicated. Cells were harvested 6 hrs later and processed for western blotting using the antibodies indicated. B and C) p21WT cell lines were treated with control siRNA or siRNA targeted to Cul4A (B) or DDB1 (C), as indicated, during parasynchronization. Cells were released and mock infected or infected with MVM at an MOI of 10. At 18 hr pi cells were treated with doxycycline to induce p21 expression. Cells were harvested at 24 hr pi and processed for western blotting using the antibodies indicated. (TIF) [file ppat.1004055.s004.tif]

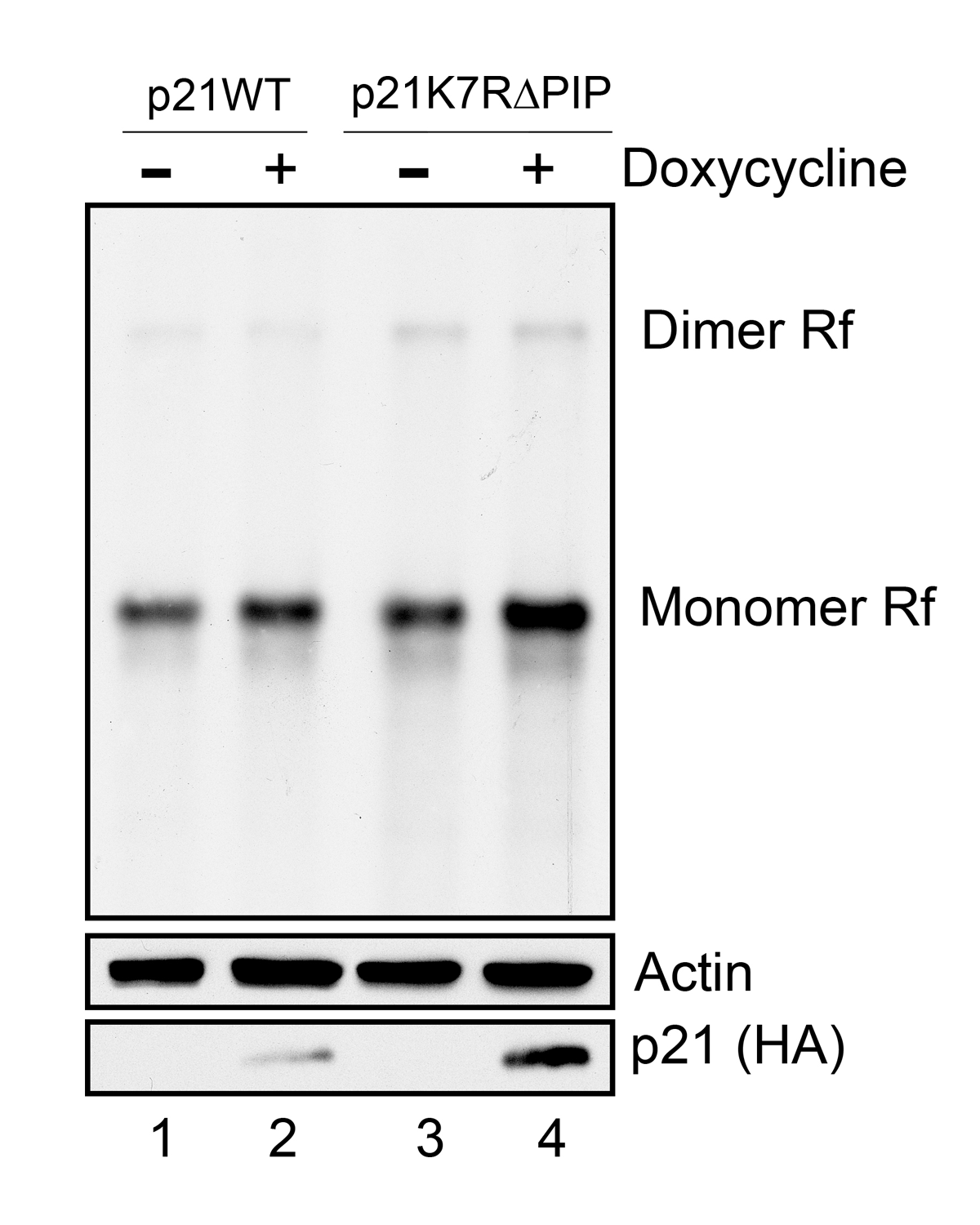

Supplement: Figure S5 — p21K7RΔPIP does not inhibit MVM replication. p21WT and p21K7RΔPIP cell lines were parasynchronized, released and infected with MVM at an MOI of 0.5. At 16 hr pi cells were treated with doxycycline to induce p21 expression and harvested 8 hrs later. Cells were processed for Southern blotting (top panel), or for western blotting using the indicated antibodies (bottom panels). (TIF) [file ppat.1004055.s005.tif]

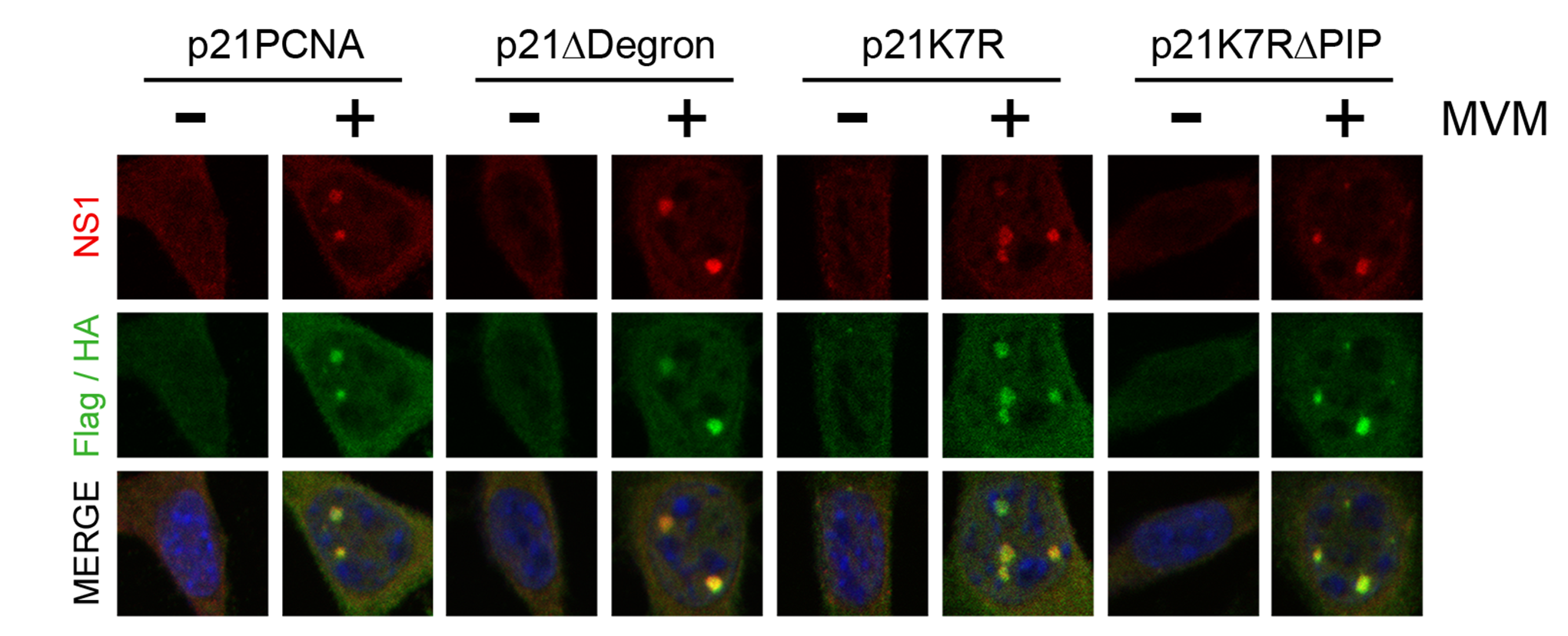

Supplement: Figure S6 — p21 mutants are recruited to MVM replication compartments. Murine A9 cell lines stably expressing FLAG-tagged p21PCNA, p21ΔDegron or HA-tagged p21K7R or p21K7RΔPIP were mock infected or infected with MVM at an MOI of 10. At 18 hr pi cells were treated with doxycycline to induce p21 expression. At 24 hr pi cells were processed for IF using antibodies against NS1 and FLAG or HA. (TIF) [file ppat.1004055.s006.tif]
